# Functional Deconstruction of AC-T Chemotherapy Reveals Interindividual Heterogeneity and Independent Drug Action in Breast Cancer

**DOI:** 10.3390/cancers18193121

**Published:** 2026-09-26

**Authors:** Lucas Damé Simões, Giulia Luiza Santos, Priscila Oliveira de Carvalho, Ernande Xavier dos Santos, Amanda Rafaela Alves Canteli, Ronaldo Morales Junior, Carolina Rossi Saccarelli, Larissa Assad João Moysés, Ligia Akemi Yamashita, Patricia Akissue de Camargo Teixeira, Thiago Yoshio Kobayashi, Vera Christina Camargo de Siqueira Ferreira, Vivian Larissa Nakamura Omura, Luma Soares de Almeida, Carlos Henrique dos Anjos, Alfredo Carlos S. D. de Barros, Anamaria Aranha Camargo, José Luiz B. Bevilacqua, Daniela G. Giannotti, Érico Tosoni Costa

**Affiliations:** 1 Molecular Oncology Center, Hospital Sírio-Libanês, São Paulo 01308-060, SP, Brazil; lucasdame@hotmail.com (L.D.S.);; 2 Núcleo de Diagnóstico por Imagem da Mama, Hospital Sírio-Libanês, São Paulo 01308-060, SP, Brazil; 3 Department of Oncology, Hospital Sírio-Libanês, São Paulo 01308-060, SP, Brazil; 4 Núcleo de Mastologia, Hospital Sírio-Libanês, São Paulo 01308-060, SP, Brazil

**Keywords:** breast cancer, patient-derived organoids, combination chemotherapy, anthracycline-taxane chemotherapy, functional precision oncology, drug response profiling, independent drug action, treatment de-escalation

## Abstract

**Background/Objectives:** Combination chemotherapy remains a cornerstone of breast cancer (BC) treatment, yet the relative contribution of individual drugs within standard regimens remains poorly understood. We investigated whether the activity of the widely used anthracycline-cyclophosphamide-taxane (AC-T) regimen reflects uniform benefit from all constituent drugs or heterogeneous drug-specific vulnerabilities across tumors. **Methods:** We combined in silico drug-response analyses, functional validation in BC cell lines, and ex vivo profiling in patient-derived organoids (PDOs). Drug responses were integrated into the Probability of Chemotherapeutic Efficacy (PCE), a multidimensional response metric combining relative drug potency, maximal efficacy, and clinically achievable free-drug exposure. To explore the broader applicability of PCE, we additionally applied it to an independent publicly available GR-based pharmacological dataset generated by the HMS LINCS Center. **Results:** Across BC models, anthracyclines and taxanes displayed only partially overlapping sensitivity profiles, indicating distinct drug-specific vulnerabilities. Functional validation in 2D cell lines and PDOs confirmed highly context-dependent responses to AC-T components. Among 21 PDOs with complete drug-response datasets, extensive interpatient heterogeneity was observed, with non-overlapping patterns of sensitivity to A, C, and T. Responses were better represented as a continuum of functional vulnerability states than as binary sensitive-versus-resistant categories. AC generated the highest proportion of favorable response profiles, with 62% of PDOs classified as partial response-like or complete response-like, compared with 48% for A, 38% for C, and 14% for T. AC activity was positively associated with the most active constituent drug within each PDO. Application of this framework to an independent public dataset produced biologically plausible response patterns consistent with the molecular characteristics of the evaluated breast cancer cell lines, supporting its computational transferability. **Conclusions:** These findings reveal substantial heterogeneity in the contribution of individual drugs to AC-T activity and are compatible with a model in which different tumors derive benefit from different regimen components. Together, these findings establish a hypothesis-generating functional framework for dissecting the contribution of individual drugs within combination chemotherapy regimens. Prospective studies correlating PDO-derived functional responses with clinical outcomes will be required to determine the predictive value and potential clinical utility of this approach before informing therapeutic decision-making.

## Simple Summary

Combination chemotherapy remains a cornerstone of breast cancer treatment, yet little is known about how much each individual drug contributes to the overall benefit observed in a given patient. In this study, we established patient-derived organoids from breast cancer biopsies and developed a clinically oriented framework to quantify the activity of individual chemotherapy agents within the widely used AC-T regimen. By integrating multiple dimensions of drug response into a single functional score, we were able to compare the relative contribution of each treatment component across tumors. We found that many tumors displayed marked asymmetry in drug sensitivity, suggesting that the benefit of combination chemotherapy may often be driven by a subset of regimen components rather than by equal activity of all drugs. These findings provide a new experimental approach for studying chemotherapy optimization and may contribute to future strategies aimed at personalizing treatment while reducing unnecessary toxicity.

## 1. Introduction

Breast cancer (BC) remains one of the leading causes of cancer-related mortality in women worldwide, and systemic therapy continues to be central to disease control in early and advanced disease [1,2]. BC comprises a biologically heterogeneous group of diseases characterized by differences in hormone receptor (HR) expression, HER2 status, tumor proliferative activity (Ki67 index), and underlying molecular alterations. Current classification integrates histopathological evaluation with ER, PR, HER2 and Ki67 assessment to define clinically distinct disease subsets, including HR+/HER2−, HER2-positive and triple-negative breast cancer (TNBC), while transcriptomic studies have further identified intrinsic molecular subtypes such as luminal A, luminal B, HER2-enriched and basal-like tumors [3,4]. This biological heterogeneity has major implications for prognosis, therapeutic response, and treatment selection, as tumors with similar clinicopathological features may exhibit markedly different drug sensitivities [5].

While targeted therapies (endocrine, HER2-targeted, antibody-drug conjugates and immunotherapy) have revolutionized BC care, chemotherapy remains a widely used treatment for a substantial proportion of patients, particularly in locally advanced and neoadjuvant settings [6]. Among standard regimens, anthracycline- and taxane-based combinations such as AC-T remain widely used because of their broad activity across clinically relevant BC contexts, although their benefits are uneven across patients and are accompanied by substantial toxicity [7]. This sequential strategy, in which AC is followed by T, is considered one of the most effective adjuvant chemotherapy approaches and has shown improved disease-free survival relative to non-taxane regimens such as AC alone, as well as to some concurrent schedules [8].

A limitation of chemotherapy development is that superiority at the population level does not necessarily imply synergistic action within each individual patient. This concept has gained renewed relevance with the paradigm of Independent Drug Action (IDA), whereby the apparent benefit of a combination may arise because different patients benefit from different drugs, rather than because the same tumor requires true pharmacological synergy [9]. Under this framework, the efficacy of combination therapy may emerge from heterogeneous drug sensitivities distributed across patients, creating multiple independent opportunities for therapeutic benefit without requiring universal synergistic interactions within each tumor. Supporting this concept, Jaaks and colleagues evaluated 2025 clinically relevant two-drug combinations across 125 molecularly characterized cancer cell lines, including 51 BC models, and found that synergy was uncommon overall (5.2%) and even less frequent in BC (4.4%) [10]. Moreover, only 27.5% of synergistic combinations were observed at both high and low anchor concentrations, highlighting the strong dose-dependence of these interactions. Most notably, chemotherapy–chemotherapy combinations showed particularly low synergy rates, with only 21 of 2337 combination-cell line pairs (0.9%) classified as synergistic, compared with 3.2% for chemotherapy-targeted and 6.1% for targeted–targeted combinations [10]. These observations challenge the routine assumption that the activity of standard polychemotherapy necessarily reflects true synergy. Instead, they suggest that regimen-level benefit may often conceal distinct single-agent vulnerabilities across tumors. This is particularly relevant in precision medicine, where molecular classification has improved therapeutic stratification but remains insufficient to identify which specific component of a standard chemotherapy regimen is driving benefit in each patient, particularly given the biological complexity generated by tumor heterogeneity and the tumor microenvironment [6,11].

Patient-derived organoids (PDOs) offer a valuable opportunity to functionally investigate patient-specific chemotherapy vulnerabilities. PDOs preserve key histopathological and molecular features of the original tumors while maintaining clinically relevant interpatient heterogeneity and enabling direct functional interrogation of drug response. Growing evidence suggests that therapeutic responses observed in PDOs may reflect clinical treatment responses, reinforcing their potential as predictive platforms for precision oncology [11,12,13,14,15]. Recent advances further highlight the ability of organoid platforms to bridge the translational gap between conventional two-dimensional cell cultures and in vivo models by preserving patient-specific biological complexity while enabling functional precision oncology approaches [16].

In BC, PDOs provide a platform not only to capture clinically relevant heterogeneity, but also to investigate how individual components of standard chemotherapy regimens contribute to treatment activity within specific tumors [17,18]. More broadly, recent benchmarking studies have emphasized the limitations of interpreting therapeutic activity using isolated pharmacological metrics or arbitrary sensitivity thresholds alone, highlighting the need for response frameworks that integrate complementary dimensions of drug activity while remaining clinically interpretable [19]. Accordingly, BC PDOs have become valuable functional platforms for evaluating interpatient variability in drug sensitivity and for exploring patient-specific therapeutic vulnerabilities under controlled experimental conditions [20].

Here, we combined in silico drug-response analyses, in vitro functional validation, and ex vivo profiling in PDOs to dissect the contribution of individual components within the standard AC-T regimen in BC. Rather than asking whether AC-T is effective as a regimen, our objective was to determine how much each constituent components contributes to its overall activity across individual tumors. Across public datasets, we first investigated whether vulnerabilities to anthracyclines and taxanes were broadly overlapping or instead distributed across distinct subsets of tumors. We then used BC cell lines to experimentally validate these dependencies under clinically anchored conditions. Finally, we established a BC-PDO platform to evaluate patient-specific responses to doxorubicin (A), 4-hydroxycyclophosphamide (C), paclitaxel (T), and the AC combination using growth-rate (GR)-corrected pharmacological metrics [21]. This step allowed us to move from population-level pharmacologic patterns to individual functional response states. To facilitate clinically oriented interpretation, drug potency, maximal efficacy, and clinically achievable exposure were integrated into a Probability of Chemotherapeutic Efficacy (PCE) framework that generates RECIST-inspired response states ranging from Progressive Disease (PD)-like to Complete Response (CR)-like profiles. Our analyses revealed extensive interpatient heterogeneity in chemotherapy response and demonstrated that distinct tumors frequently rely on non-overlapping pharmacological vulnerabilities. Although AC generally produced the most favorable response profiles, the dominant contributor to treatment activity varied substantially across PDOs. Consistent with an IDA-like framework, responses to combination therapy were often associated with the activity of individual components rather than universal synergy between them. Together, these analyses establish a hypothesis-generating framework for investigating how individual drugs contribute to the activity of standard polychemotherapy and provide a functional rationale for future studies evaluating whether patient-specific drug vulnerabilities could support chemotherapy personalization.

## 2. Materials and Methods

### 2.1. In Silico Analysis of Drug Sensitivity in GDSC BC Models

Public data were retrieved from the Genomics of Drug Sensitivity in Cancer (GDSC) resource, using the GDSC2 release as the primary source for BC cell-line drug-response profiles [22]. To model the AC-T regimen and its clinically relevant alternatives, we extracted single-agent IC_50_ values for doxorubicin (DOXO), cyclophosphamide (CICLO), paclitaxel (PTX), epirubicin (EPI), and docetaxel (DTX). These agents were selected to represent the anthracycline, cyclophosphamide, and taxane backbone of AC-T, together with structurally related drugs commonly used in BC treatment. Cyclophosphamide was analyzed *in silico* because it is the compound available in GDSC, whereas all functional experiments employed its principal active metabolite, 4-hydroxycyclophosphamide (4-OHC, Merck, Darmstadt, Germany), to model activated drug exposure in *ex vivo* and *in vitro* systems lacking hepatic metabolism. After preprocessing, the final analyzable panel comprised 50 BC cell lines representing the major molecular subtypes included in this study. The GDSC database and associated cell model annotations are publicly accessible through the GDSC portals. To place in vitro sensitivity into a clinically meaningful framework, IC_50_ values were compared with clinically achievable free plasma concentrations (Cmax) obtained from the literature [23]. Cell lines with IC_50_ values below Cmax were considered potentially responsive, whereas those with IC_50_ values above Cmax were considered likely non-responsive. To reduce ambiguity around the clinical threshold and increase contrast for comparative analyses, we additionally applied a distance-based subsampling strategy centered on Cmax. Specifically, for each drug, the 70% lowest IC_50_ values were classified as responsive and the 70% highest IC_50_ values as non-responsive, whereas models within ±30% of Cmax were considered indeterminate. This approach was used to strengthen subgroup contrasts in histogram- and correlation-based analyses while preserving the clinically anchored logic of the classification framework. Cyclophosphamide served as an internal pharmacologic control for this framework. For pairwise drug comparisons, IC_50_ correlations between anthracyclines (DOXO, EPI), taxanes (PTX, DTX), and cross-class combinations were evaluated using Spearman’s rank correlation. Statistical analyses and graphical visualization were performed in GraphPad Prism version 9.2 (GraphPad Software, San Diego, CA, USA) and RStudio version 2026.01.1+403 (R Foundation for Statistical Computing, Vienna, Austria).

### 2.2. BC Cell Lines and Cultivation

BT-474 and MDA-MB-231 BC cell lines were used as 2D models for functional validation of chemotherapy response. BT-474, a HER2-positive luminal B model, was obtained from the American Type Culture Collection (ATCC, Manassas, VA, USA) and MDA-MB-231, a triple-negative/basal-like model, was also obtained from ATCC. Cells were cultured in RPMI-1640 (Sigma-Aldrich, St. Louis, MO, USA) supplemented with 10% fetal bovine serum (FBS) and 1% L-glutamine (both from Gibco™, Thermo Fisher Scientific, Waltham, MA, USA). Both cell lines were maintained at 37 °C in a humidified atmosphere containing 5% CO_2_ and routinely passaged upon reaching 80–90% confluence. All cultures were tested for Mycoplasma contamination using PCR-based detection, and only negative cultures were used for experiments. Cell line identity was verified by STR-based confirmation using the GenePrint 10 System (Promega, Madison, WI, USA).

### 2.3. MTT-Based Cell Viability Assays

BT-474 and MDA-MB-231 cells were seeded in 96-well plates at a density of 8000 cells per well and allowed to adhere for 24 h before treatment. Cells were then exposed to the indicated drugs for 72 h in MDA-MB-231 and 120 h in BT-474, reflecting the longer doubling time of BT-474 cells under the culture conditions used. The compounds tested in this section included the main components of the AC-T regimen: doxorubicin (DOXO, A), 4-hydroxycyclophosphamide (4-OHC, C), and paclitaxel (PTX, T), as well as the clinically related agents epirubicin (EPI) and docetaxel (DTX) (all from Merck, Darmstadt, Germany). Combination experiments using AC (DOXO plus 4-OHC) were performed using the same concentration ranges adopted for single-agent treatments. Drug concentrations were selected to span clinically relevant exposure ranges, based on Cmax, in order to maximize translational relevance and avoid suprapharmacologic conditions of limited clinical interpretability [23]. At the end of treatment, MTT reagent was added to each well and incubated according to standard procedures. Formazan crystals were then solubilized, and absorbance was measured in a microplate reader. Cell viability values were normalized to vehicle-treated controls and subsequently used for dose–response analysis. Drug-response metrics were calculated using the GR framework, including GR_50_ and GR_max_, as described [20].

### 2.4. Growth-Rate Analysis and PCE-Based Classification of Drug Response

Drug-response profiles were quantified using growth-rate (GR) metrics, thereby allowing more robust comparisons across models with distinct doubling times [21]. GR metrics were selected because they minimize confounding effects arising from differences in cellular proliferation rates and provide a more reliable framework for comparing drug activity across heterogeneous biological models. From GR dose-response curves, we extracted GR_50_ and GR_max_ as the primary measures of drug potency and maximal effect, respectively. Throughout the study, these parameters were interpreted relative to the clinically achievable free plasma concentration (Cmax free) of each compound. For simplicity, Cmax refers throughout this manuscript to the clinically achievable free plasma concentration (Cmax free), unless otherwise specified. To facilitate clinically oriented interpretation of PDO drug-response profiles, we developed an exploratory multidimensional metric termed Probability of Chemotherapeutic Efficacy (PCE). PCE integrates GR_50_, GR_max_, and Cmax into a single continuous score ranging from 0 to 100.

PCE was calculated according to the following equation:

$$PCE=100\times \left(\frac{Cmax\mathrm{free}}{Cmax\mathrm{free}+\;GR50}\right)\times \left(\frac{1-GRmax}{2}\right)$$

The metric was designed to facilitate comparative interpretation of multidimensional pharmacological response profiles while preserving information provided by GR analyses. PCE was developed here for comparative analyses within the present study and should not be interpreted as a clinically validated predictor of therapeutic response. The first component of the equation quantifies the relationship between Cmax and drug potency (GR_50_), whereas the second component incorporates maximal efficacy through a normalized transformation of GR_max_ values. Equal weighting was intentionally adopted to provide a simple, biologically interpretable, and readily transferable framework integrating two complementary pharmacological properties (GR_50_ and GR_max_), while avoiding empirically optimized coefficients that would require independent model training and external calibration.

For descriptive purposes, PCE values were subsequently grouped into RECIST-inspired pharmacological response categories [24]: scores below 20 were classified as Progressive Disease-like (PD-like), scores from 20 to 49 as Stable Disease-like (SD-like), scores from 50 to 89 as Partial Response-like (PR-like), and scores ≥ 90 as Complete Response-like (CR-like). These categories do not represent radiological RECIST classifications, but rather predefined in vitro response strata derived from the combined relationship between GR_50_, GR_max_, and Cmax.

### 2.5. External Computational Application of the PCE Framework

To evaluate the general applicability of the PCE framework, we applied the algorithm to an independent publicly available GR-based pharmacological dataset generated by the HMS LINCS Center [25]. This dataset comprises dose–response experiments performed in six human breast-derived cell lines (five BC cell lines and one non-malignant mammary epithelial cell line), in which cell numbers were quantified 72 h after treatment with 107 small-molecule compounds using the same GR formalism adopted throughout the present study. GR_50_ and GR_max_ values were obtained directly from the processed dataset. PCE values were subsequently calculated and pharmacokinetic assumptions adopted throughout the present study. Because calculation of PCE requires Cmax, only compounds with available human pharmacokinetic data could be included, resulting in 51 independent drug–cell line experiments suitable for analysis. Human pharmacokinetic parameters were obtained from ref. [23], and free plasma concentrations were estimated from total Cmax and plasma protein binding.

### 2.6. Study Cohort and BC Tissue Acquisition

Patients were eligible for inclusion if they presented breast lesions classified as BI-RADS 4C or 5 and were referred for clinically indicated biopsy. All participants provided written informed consent prior to sample collection. The study was conducted in accordance with the Declaration of Helsinki and approved by the Research Ethics Committee of Hospital Sírio-Libanês (CAAE 68346623.1.0000.5461). Fresh breast tissue samples were prospectively collected from treatment-naïve patients between 1 September 2024 and 8 January 2026, and processed under sterile conditions, typically within 2 h of collection. A total of 50 biopsies were obtained, including 47 malignant and 3 benign lesions. Using this workflow, 29 long-term BC-PDO cultures were established across diverse molecular subtypes, histological patterns, and tumor grades. Detailed clinicopathological characteristics of the PDOs included in the functional analyses are summarized in Supplementary Table S2.

### 2.7. Patient-Derived Organoid (PDO) Cultures and Drug Screening

PDO cultures were maintained in a defined, serum-free medium supplemented with factors required for breast epithelial stem cell maintenance and proliferation, as previously described by [17]. Briefly, fresh breast specimens were mechanically and enzymatically dissociated and embedded in basement membrane extract (Cultrex; R&D Systems, Minneapolis, MN, USA) as dome cultures in multiwell plates. PDOs were maintained in BC medium: Advanced DMEM/F12 supplemented with 100 U/mL penicillin/streptomycin, 10 mM HEPES, GlutaMAX, 1× B27 supplement, 1.25 mM N-acetyl-L-cysteine, 10 mM nicotinamide, 5 μM Y-27632, 5 nM heregulin β-1, 500 nM A83-01, 5 ng/mL epidermal growth factor (EGF), 20 ng/mL fibroblast growth factor 10 (FGF10), 100 ng/mL Noggin, 250 ng/mL R-spondin 1, 500 nM SB202190, 5 ng/mL FGF7 and 0.05 mg/mL Primocin. Cultures were maintained at 37 °C in a humidified atmosphere containing 5% CO_2_, and medium was replaced every 3–4 days. PDOs were passaged by mechanical disruption and/or enzymatic dissociation when cultures reached sufficient density and structural complexity for continued expansion. PDO establishment was considered successful when long-term expanding cultures could be maintained for at least 4 serial passages. Morphology was monitored by bright-field microscopy throughout culture, and representative lines were subsequently processed for histopathological and immunophenotypic comparison with the corresponding parental tumors. Drug response was assessed using CellTiter-Glo^®^ 3D Cell Viability Assay (Promega, Madison, WI, USA) and analyzed using GR metrics as described in ref. [20]. Drug-response experiments were performed using biological replicates whenever material availability permitted, with three to four technical replicates per condition.

### 2.8. CellTiter-Glo 3D Drug-Response Assay in BC-PDOs

For drug-response assays, BC-PDOs were dissociated and replated in 96-well plates at a density of 1500–4000 cells embedded in 5 µL of BME (Cultrex, R&D Systems) in BC medium. After recovery and re-establishment in 3D culture, PDOs were exposed to five 4-fold spaced concentrations of the indicated compounds for 4 days. For the chemotherapeutic agents analyzed in this study, five concentrations were tested across a clinically anchored range, with the highest dose set at 4-fold above the reported free plasma Cmax and each subsequent concentration generated by 4-fold serial dilution. Cell viability was quantified using the CellTiter-Glo^®^ 3D Cell Viability Assay, according to the manufacturer’s instructions (Promega, Madison, WI, USA). Briefly, after 4 days of treatment, plates were equilibrated to room temperature and CellTiter-Glo 3D reagent was added in a volume equivalent to the culture medium present in each well. Plates were shaken for 5 min to promote cell lysis and ATP release, followed by a 25 min incubation at room temperature to stabilize the luminescent signal. Cell viability was measured in black, flat-bottom 96-well polystyrene microplates (Corning Inc., Corning, NY, USA). Dose–response curves were generated from normalized luminescence values and subsequently analyzed in R script using the GR framework [20], allowing extraction of GR_50_ and GR_max_ for each PDO–drug pair.

### 2.9. Statistical Analysis

Statistical analyses were performed in GraphPad Prism (version 9.20) and R (version 4.4.2). For GDSC-based analyses, IC_50_ values were analyzed on a log_10_ scale, distributions were visualized with histograms and density overlays, and pairwise drug-response associations were assessed using Spearman’s rank correlation. Categorical comparisons of sensitivity and resistance were based on contingency tables, from which odds ratios and *p* values were derived, with false discovery rate (FDR) correction applied where appropriate. For 2D cell-line and PDO assays, dose–response curves were analyzed using the GR framework [21], and GR_50_ and GRmax were extracted by nonlinear regression. When 50% growth-rate inhibition was not achieved within the tested concentration range, GR_50_ was reported as infinity (GR_50_ = Inf), as recommended for GR analyses. All statistical tests were two-sided, and *p* values < 0.05 were considered statistically significant. Because this study is primarily descriptive and hypothesis-generating, statistical analyses were designed to characterize drug-response heterogeneity rather than to establish predictive clinical models.

### 2.10. Use of Generative Artificial Intelligence

Generative artificial intelligence tools were used during manuscript preparation to assist in the creation of conceptual graphical elements, including workflow schematics, as well as for language editing, grammar correction and formatting of the text. All scientific content, analyses, graphs, interpretations, and conclusions were conceived, reviewed, and validated by the authors.

## 3. Results

### 3.1. Public GDSC Drug-Response Data Reveal Heterogeneous Dependence on Individual AC-T Components in Breast Cancer

To determine whether heterogeneous sensitivity to individual AC-T components is already evident across BC models, we first interrogated public drug-response data from the Genomics of Drug Sensitivity in Cancer (GDSC) pharmacogenomic resource [14]. We analyzed a panel of 50 BC cell lines comprising the major molecular subtypes and evaluated their response to the agents of the AC-T regimen: an A, usually doxorubicin (DOXO), cyclophosphamide (CICLO), and a T, usually paclitaxel (PTX). We also included the related compounds epirubicin (EPI) and docetaxel (DTX).

As an initial approximation of clinical activity, sensitivity was defined using a simplified framework based on the relationship between drug potency (IC_50_) and achievable free plasma concentrations (Cmax) [23], whereby models with IC_50_ values below Cmax were considered potentially responsive and those above Cmax were considered likely non-responsive. As shown in Figure 1A–E, IC_50_ values followed a continuous distribution across all tested agents, supporting a model in which chemosensitivity is heterogeneous rather than dichotomous.

Density curves overlaid on the histograms in Figure 1A–E were largely unimodal, suggesting that variability arose predominantly from gradual gain in drug sensitivity rather than sharply segregated subpopulations. To reduce ambiguity around the clinical threshold, we applied a distance-based subsampling strategy, retaining the 70% lowest IC_50_ values as responsive, the 70% highest as non-responsive, and models within ±30% of Cmax (indicated as a red vertical lines in Figure 1A–E) as indeterminate.

Within this criterion, among anthracyclines (A) class, DOXO displayed a predominantly sensitive profile (72% sensitive, 10% intermediate, 18% resistant), whereas EPI showed a broader distribution with increased resistance (51% sensitive, 14% intermediate, 34% resistant) (Table 1). This difference was statistically significant (FDR-adjusted *p* = 0.04), corresponding to an approximately 2.4-fold higher likelihood of response for DOXO compared with EPI (Supplementary Table S1). Among taxanes (T) class, PTX showed the highest activity (78% sensitive, 11% intermediate, 11% resistant), followed by DTX (64% sensitive, 11% intermediate, 25% resistant) (Table 1). Although PTX showed a higher proportion of sensitive models, this difference did not reach statistical significance (FDR-adjusted *p* = 0.24), consistent with partially overlapping activity profiles. Importantly, the relatively small but consistent intermediate fraction (10–14%) across drugs highlights the continuous nature of IC_50_ distributions and underscores the importance of incorporating uncertainty margins when translating in vitro drug sensitivity into clinically meaningful classifications. In contrast, CICLO showed IC_50_ values consistently above Cmax, with 100% of models classified as resistant, consistent with its requirement for metabolic activation and therefore serving as an internal calibration control for our clinically anchored framework (Table 1).

We next asked whether the activity of the AC-T regimen could be decomposed into drug-specific sensitivities, testing whether individual models preferentially depended on one component of the regimen rather than exhibiting uniform cross-drug responsiveness. Because CICLO cannot be directly evaluated *in vitro*, correlation analyses of IC_50_ profiles across the remaining agents were used to assess intra- and inter-class concordance (Figure 1F–I and Supplementary Table S1). Within the T class, PTX and DTX responses showed moderate correlation (r = 0.53, *p* = 0.0009) (Figure 1F), with 61% of models sensitive to both agents, 30% discordant, and 9% resistant to both. Consistent with this, pairwise analysis showed a trend toward higher sensitivity for PTX than DTX (odds ratio 1.98), although this did not remain significant after multiple-testing correction (FDR-adjusted *p* = 0.24; Supplementary Table S1). Anthracyclines (DOXO and EPI) showed a similar pattern (Figure 1G), with 54% concordant sensitivity, 34% discordance, and 12% dual resistance. Here, DOXO exhibited a significantly higher proportion of sensitive models than EPI (odds ratio 2.4; FDR-adjusted *p* = 0.04, Supplementary Table S1).

Inter-class comparisons revealed even greater divergence. For DOXO versus PTX (Figure 1H), only 33% of models were sensitive to both agents, whereas 44% showed discordant responses and 22% were resistant to both, indicating substantial non-overlapping vulnerabilities. A similar pattern was observed for DOXO versus DTX (Figure 1I), with 47% concordant sensitivity, 41% discordance, and 12% dual resistance. Consistently, PTX showed a significantly higher likelihood of response than EPI (odds ratio 3.3; FDR-adjusted *p* = 0.02; Supplementary Table S1), reinforcing the presence of drug-specific dependencies across classes. Notably, 30–44% of BC cell lines displayed discordant responses, defined as sensitivity to one agent but resistance to another, either within the same class or across classes (Figure 1F–I, upper-left and lower-right quadrants; Supplementary Table S1). This large discordant fraction identifies non-overlapping therapeutic vulnerabilities across these models.

Together, these *in silico* analyses indicate that BC models do not respond uniformly to the components of AC-T, but instead display partially overlapping, drug-specific sensitivities consistent with an IDA framework. We therefore next asked whether these non-overlapping dependencies could be reproduced experimentally in representative 2D BC cell lines.

### 3.2. BC Cell Lines Validate Non-Overlapping Dependencies on AC-T Components

To experimentally validate the non-overlapping drug dependencies inferred from the GDSC analysis, we next quantified chemotherapy response in two 2D BC cell lines using growth-rate (GR)-based metrics, which normalize drug effects to proliferation kinetics and thus minimize confounding differences in baseline doubling time under identical culture conditions [20]. We selected MDA-MB-231, a triple-negative/basal-like model, and BT-474, a HER2-positive luminal B model, to capture biologically distinct BC settings. Cells were exposed to the main components of the AC-T regimen, using 4-OHC as the active metabolite of cyclophosphamide, and responses were quantified by GR_50_ and GR_max_. Consistent with the *in silico* analysis, the two models displayed clearly divergent dependencies on individual AC-T components (Figure 2A,B and Table 2).

In both cell lines, 4-OHC was inactive under the tested conditions, failing to achieve measurable growth-rate inhibition within the concentration range evaluated (GR_50_ = inf; GR_max_ = 1.00). This finding was consistent across molecular backgrounds and suggested that the cyclophosphamide component displayed limited activity under these experimental conditions.

In MDA-MB-231 cells, therapeutic vulnerability was largely restricted to the T arm. Both PTX (GR_50_ = 0.0025 µM; GR_max_ = −0.40) and DTX (GR_50_ = 0.0028 µM; GR_max_ = −0.59) remained active at concentrations substantially below clinically achievable free exposure levels, indicating robust pharmacological activity. In contrast, DOXO displayed limited potency and failed to induce cytotoxicity (GR_50_ = 1.35 µM; GR_max_ = 0.23), while EPI showed intermediate activity (GR_50_ = 0.74 µM; GR_max_ = −0.69). Importantly, the response profile of DOXO + 4-OHC closely resembled that of DOXO monotherapy (GR_50_ = 1.40 µM; GR_max_ = 0.33), indicating no evident functional gain from addition of 4-OHC to the AC backbone (Figure 2B). Thus, within this model, biologically relevant activity was concentrated predominantly within the taxane component of the regimen.

By contrast, BT-474 cells exhibited a broadly chemosensitive phenotype. Although 4-OHC again remained inactive, both anthracycline- and taxane-based agents produced substantial growth inhibition. PTX and DTX were highly potent and fully cytotoxic (GR_max_ = −1.00), whereas DOXO also demonstrated marked activity (GR_50_ = 0.08 µM; GR_max_ = −1.00). EPI remained active but achieved a less pronounced maximal effect (GR_max_ = −0.46). Notably, however, addition of 4-OHC failed to improve the pharmacological profile of DOXO, with the AC combination producing essentially identical GR_50_ and GR_max_ values to anthracycline monotherapy (Figure 2B). These findings indicate that BT-474 was sensitive to both anthracycline and taxane classes, but not to the cyclophosphamide surrogate.

Beyond the specific drug-response patterns observed, these experiments highlighted an important conceptual limitation of interpreting chemotherapy activity through any single pharmacological parameter. Potency measurements alone may underestimate compounds capable of achieving deep cytotoxic responses, whereas maximal efficacy alone fails to account for whether such effects occur within clinically achievable exposure ranges. As recently emphasized by multidimensional drug-evaluation frameworks, clinically meaningful activity emerges from the integrated relationship between potency, maximal effect, and therapeutic exposure rather than from isolated metrics considered independently. Consistent with this view, the present data revealed distinct pharmacological states that could not be fully resolved by simple sensitive-versus-resistant classifications.

Taken together, these results demonstrate that vulnerabilities to AC-T components are highly context-dependent and unevenly distributed across BC models. The lack of functional superiority of AC over DOXO alone further suggests that regimen-level activity may emerge from dominant contributions of specific constituent drugs rather than uniform benefit from all regimen components. These observations motivated the development of a multidimensional response framework for PDO analyses, integrating potency, maximal efficacy, and clinically achievable exposure into a clinically oriented assessment of chemotherapeutic activity.

### 3.3. Patient-Derived Organoids Reveal Interindividual Heterogeneity and Non-Overlapping Vulnerabilities Within AC-T Chemotherapy

To investigate whether the non-overlapping drug dependencies observed in GDSC datasets and 2D tumor cell lines were preserved at the patient level, we established a BC-PDO platform from fresh tumor biopsies obtained from treatment-naïve patients at our institution (Figure 3). The experimental workflow is shown in Figure 3A. A total of 50 biopsies were collected, of which 47 (94%) corresponded to malignant lesions and three (6%) to benign lesions (Figure 3B). Among the 47 malignant biopsies, molecular subtype information was available for 46 tumors, comprising 24 HR+/HER2− (52%), six TNBC (13%), three HER2-positive (6.5%), one triple-positive (2%), and 12 cases (26%) pending complete molecular classification.

Using optimized culture conditions, 29 long-term BC-PDO models were successfully established and maintained. Of these, 21 generated complete GR-based response profiles for DOXO (A), 4-OHC (C), PTX (T), and the AC combination, enabling direct comparison of patient-specific vulnerabilities across the AC-T regimen. PDOs displayed diverse morphologies, including compact solid structures and grape-like architectures, while representative models retained key histopathological and immunophenotypic features of their parental tumors, including expression of ER, PR, HER2, and Ki67 (Figure 3C). Collectively, these observations support the use of BC-PDOs as patient-proximal functional models for investigating chemotherapy response heterogeneity.

PDOs were subsequently exposed to clinically relevant concentration ranges of A, C, T, or the AC combination. Drug activity was quantified using GR metrics (GR_50_ and GR_max_), which account for differences in baseline proliferation rates among PDOs [13]. To facilitate clinically oriented interpretation, these parameters were further integrated with achievable free plasma exposure through the Probability of Chemotherapeutic Efficacy (PCE), a RECIST-like framework that combines GR_50_, GR_max_, and Cmax into a single response score ranging from 0 to 100. Instead of dichotomizing PDO responses as sensitive or resistant based on a single pharmacological parameter, we integrated potency, maximal effect, and clinically achievable exposure into a continuous response framework.

Application of the PCE framework immediately revealed extensive functional heterogeneity across the cohort (Figure 3D). Because PCE scores were interpreted using RECIST-inspired response states, PDOs could be classified as PD-like (PCE < 20), SD-like (20 ≤ PCE < 49), PR-like (50 ≤ PCE < 89), or CR-like (PCE ≥ 90), reflecting progressively more favorable predicted response profiles. Hierarchical clustering identified distinct functional patterns, including PDOs with broadly favorable response profiles across multiple treatments (e.g., HSL-PDO7, HSL-PDO17, HSL-PDO19), PDOs displaying predominantly unfavorable response states (e.g., HSL-PDO9, HSL-PDO10, HSL-PDO12, HSL-PDO13), and intermediate phenotypes characterized by selective responsiveness to specific components of the AC-T regimen (e.g., HSL-PDO20, HSL-PDO16 and HSL-PDO15). Importantly, these response patterns were frequently non-overlapping: several PDOs classified as PR-like or CR-like for one specific treatment displayed SD-like or PD-like profiles for others, indicating that chemotherapy sensitivity was highly individualized and could not be inferred uniformly across patients. This observation is consistent with the concept that distinct tumors may rely on different pharmacological vulnerabilities even when exposed to the same standard-of-care regimen.

### 3.4. A RECIST-like PCE Framework Reveals a Continuum of Chemotherapy Response States Across BC-PDOs

The clinicopathological characteristics of the 21 BC-PDOs included in all downstream pharmacological analyses are summarized in Supplementary Table S2. This analytical subset remained enriched for HR+/HER2− tumors (12/21), whereas HER2-positive (HSL-PDO#19, 1/21) and TNBC (HSL-PDO#8, #12, #16, 3/21) PDOs were comparatively underrepresented. To facilitate biological interpretation of the heterogeneous pharmacological responses observed across PDOs, we used the PCE framework to translate these continuous PDO-derived drug-response measurements into clinically interpretable RECIST-like response states. Consistent with the extensive functional heterogeneity observed in the Figure 3D, all treatment conditions displayed broad distributions of PCE values, indicating substantial interpatient variability in both treatment sensitivity and maximal therapeutic effect. Among all treatment conditions, AC exhibited the highest overall response profile (median PCE = 64.3), followed by doxorubicin (median = 49.1), whereas 4-OHC (median = 28.9) and PTX (median = 10.9) displayed substantially lower central tendencies. Importantly, each treatment spanned a wide range of PCE values, extending from PD-like to CR-like states, highlighting the coexistence of highly responsive and poorly responsive PDOs within the same cohort (Figure 4A). Notably, PTX exhibited a pronounced enrichment of low-scoring profiles, whereas AC and DOXO were associated with a greater frequency of high-response states.

To further translate these continuous response distributions into clinically interpretable categories, PCE scores were grouped into RECIST-like response states (Figure 4B). Marked differences emerged between treatment conditions. AC generated the highest proportion of favorable response states, with 62% of PDOs classified as PR-like or CR-like, including 24% categorized as SD-like. DOXO also produced favorable responses in nearly half of the cohort (48%), whereas 4-OHC yielded PR-like or CR-like states in 38% of PDOs. In contrast, PTX exhibited predominantly unfavorable response profiles, with 67% of PDOs classified as PD-like and only 14% reaching PR-like states, while no PDO achieved a CR-like response (Figure 4B). Despite the predominance of PD-like profiles, marked interpatient variability was observed, with strong PTX responses identified in selected TNBC (HSL-PDO#8 and #16) and HER2-positive (HSL-PDO#19) PDOs (Figure 3D). Notably, favorable responses (PR- and CR-like) were observed for all treatment conditions, whereas no single treatment produced uniformly favorable outcomes across the entire cohort. Together, these findings indicate that chemotherapy responsiveness is distributed along a continuum of response states rather than a binary sensitive-versus-resistant pattern and further support the existence of substantial interpatient heterogeneity in vulnerability to the individual components of the AC-T regimen.

Representative dose–response curves further illustrated the non-overlapping response phenotypes observed across the PDO cohort (Figure 4C, all dose–response curves are provided in Supplementary Figure S1). HSL-PDO3 displayed a highly favorable response to AC (PCE = 94.1; CR-like), despite only modest activity of 4-OHC alone (PCE = 31.4; SD-like) and complete lack of PTX activity (PCE = 0.0; PD-like). In contrast, HSL-PDO4 exhibited strong responses to both doxorubicin (PCE = 97.9; CR-like) and AC (PCE = 90.4; CR-like), while remaining essentially unresponsive to 4-OHC (PCE = 0.1; PD-like). These representative examples demonstrate that PDOs can achieve similar overall levels of regimen responsiveness through fundamentally different patterns of drug vulnerability, reinforcing the concept of non-overlapping pharmacological dependencies among patients.

Finally, to investigate whether AC activity was primarily determined by the most active constituent drug, we compared AC PCE scores with the highest PCE achieved by either A or C in each PDO (Figure 4D). AC responses were positively associated with the best constituent response (Spearman ρ = 0.64, *p* < 0.0001), indicating that PDOs highly responsive to at least one component of the combination generally tended to exhibit favorable AC responses. However, substantial dispersion around the identity line was observed, with several PDOs displaying AC activities that deviated markedly from those predicted by the most active single agent. These findings suggest that although the dominant constituent drug contributes substantially to combination efficacy, AC responses cannot be fully explained by simple constituent dominance alone. Rather, the data support a model in which both drug-specific effects and combination-dependent interactions contribute to the observed response landscape.

Next, to further examine the biological plausibility and external applicability of the PCE framework, we applied it to an independent GR-based pharmacological dataset generated by the HMS LINCS Center [25]. Of the 107 compounds initially available, only drugs with corresponding human pharmacokinetic information required for Cmax estimation were eligible, yielding 51 independent drug–cell line experiments. As expected from their target biology, most targeted agents displayed predominantly PD-like response states, whereas the broad-spectrum cytotoxic agent mitoxantrone exhibited substantially higher PCE values across multiple models (Supplementary Figure S2). Notably, dasatinib produced the highest PCE score among all evaluated breast cancer cell lines in the TNBC model MDA-MB-231 (PCE = 23.3; SD-like), whereas HER2-positive SK-BR-3 remained PD-like (PCE = 0.2), consistent with previous studies describing MDA-MB-231 as relatively sensitive and SK-BR-3 as resistant to dasatinib [26]. The successful application of PCE to an independent publicly available GR-based dataset further suggests that the framework is analytically transferable beyond the present experimental platform, although prospective clinical validation remains essential.

Importantly, these observations were obtained across three independent experimental levels, public pharmacogenomic datasets, conventional 2D cell lines, and patient-derived organoids, supporting the robustness of the observed pattern of heterogeneous constituent-drug vulnerabilities. The PCE framework enabled integration of potency, maximal efficacy, and clinically achievable exposure into a unified response metric, revealing substantial interpatient variation in both the magnitude and pattern of chemotherapy vulnerability.

## 4. Discussion

Here, we functionally dissected the AC-T regimen across public pharmacogenomic datasets, conventional 2D models, and PDOs. Across all experimental systems, individual drugs contributed unequally to overall chemotherapy activity, supporting a model in which regimen efficacy emerges from heterogeneous, context-dependent vulnerabilities rather than universal benefit from every constituent drug. This interpretation is biologically plausible in BC, a disease characterized by extensive intertumoral and intratumoral heterogeneity arising from diverse genetic, epigenetic, and transcriptomic determinants, together with dynamic cellular plasticity [3,4,5]. Even tumors classified within the same clinical subtype can exhibit substantial functional divergence, limiting the predictive value of subtype labels alone for chemotherapy selection [27]. Our PDO data fit well within this framework. Although the cohort was enriched for HR+/HER2− tumors (52%), responses to the individual components of AC-T remained highly variable, with PDOs spanning a broad continuum of functional states. These findings reinforce the notion that clinically relevant therapeutic vulnerabilities cannot be inferred reliably from clinicopathologic classification alone. In this sense, PDOs do not merely recapitulate morphology or marker expression, but also expose patient-specific drug sensitivities that remain obscured in population-level analyses.

A central implication of our findings is that IDA provides a plausible framework for interpreting AC-T activity. In the metastatic setting, Plana et al. (2022) proposed that many successful oncology drug combinations may operate through a “bet-hedging” mechanism, whereby different patients benefit from different agents and the population-level efficacy of the combination emerges from multiple independent opportunities for response rather than from pharmacological synergy within the same tumor [9]. Our findings are broadly consistent with this interpretation, emerging independently from three complementary experimental levels (public pharmacogenomic data, conventional cell lines, and PDOs), strengthening the biological consistency of the observed pattern. Across PDOs, favorable response states frequently arose through distinct drug-specific vulnerabilities, and AC responses were positively associated with the most active constituent agent. At the same time, the relationship was not perfect, indicating that AC activity is not reducible to simple constituent dominance alone, but rather reflects partially independent drug effects together with context-dependent interactions. We therefore interpret our data as being functionally compatible with an IDA-like model, while avoiding the stronger claim that synergy is absent in all tumors.

This view is also consistent with large-scale pharmacologic interaction studies. Jaaks et al. (2022), in a systematic analysis of 2025 clinically relevant two-drug combinations across 125 molecularly characterized cancer cell lines, reported that synergy was uncommon overall (5.2%) and even less frequent in breast cancer (4.4%) [10]. Chemotherapy–chemotherapy combinations showed particularly low synergy rates, with only 0.9% of combination–cell line pairs classified as synergistic. Collectively, these findings challenge the assumption that the efficacy of standard polychemotherapy necessarily reflects widespread pharmacologic synergy and instead support the possibility that much of the observed benefit may derive from heterogeneous, partially independent drug sensitivities.

Our results also help explain why standard combination therapy can appear broadly effective at the population level while still containing components that may add little benefit for individual patients. In our PDO cohort, 76% of tumors were classified as sensitive to the complete AC-T regimen, yet only 28% of all PDOs showed evidence consistent with supra-additive interaction between A and C, whereas in most cases AC activity could be explained largely by the dominant contribution of a single agent. This distinction is clinically consequential because it suggests that some patients may be exposed to toxic drugs that contribute little to the functional response of their tumor. In that context, functional precision oncology is not only a strategy to identify active regimens, but also a framework to rationally deconstruct empiric polychemotherapy, prioritize the truly active component, and reduce unnecessary toxicity.

This concept is supported by clinical data from anthracycline de-escalation studies. In the phase III PlanB and SUCCESS C trials, anthracycline-containing regimens did not improve DFS or OS over six cycles of docetaxel plus cyclophosphamide in most patients with intermediate- to high-risk HER2-negative early BC, while producing more grade 3–4 adverse events. Although these trials were not designed to test IDA directly, they are compatible with the possibility that a substantial fraction of patients derive limited benefit from anthracycline exposure. Our findings provide an exploratory biological framework for interpreting such observations and suggest that functional response profiling may provide a framework for identifying patients in whom regimen simplification or selective omission of specific components could be prospectively evaluated in future clinical studies. in appropriately selected patients. More broadly, this aligns with the growing adoption of response-adapted treatment strategies in early BC, such as PHERGain [28], TRAIN-3 [29], and I-SPY2.2 [30]. Unlike these approaches, which adapt treatment after therapy has already begun, PDO-based profiling offers the theoretical advantage of identifying component-level vulnerabilities before treatment initiation. Whether such ex vivo deconvolution can be translated into safe clinical de-escalation strategies, however, remains to be determined. Prospective studies correlating PDO-derived functional response profiles with pathological response and long-term clinical outcomes will be required to establish the predictive value and clinical utility of this approach. Recent advances in organoid-based translational research, together with emerging clinical validation frameworks and ongoing prospective clinical trials evaluating PDO-guided treatment strategies, support the feasibility of prospectively validating PDO-guided functional profiling in precision oncology, although this remains to be formally demonstrated in BC [16,31,32,33].

To facilitate interpretation of these multidimensional response patterns, we introduced the PCE framework as an auxiliary translational layer. Rather than replacing conventional pharmacological metrics such as GR_50_ or GR_max_, PCE integrates potency, maximal effect, and clinically achievable exposure into a single clinically anchored response summary. Importantly, RECIST-like categories derived from PCE should not be interpreted as direct clinical outcome predictions. Instead, they provide a standardized language for mapping continuous pharmacologic responses onto clinically familiar response states. Accordingly, PCE should be viewed as an interpretive framework rather than the principal conceptual contribution of the present study.

Several limitations should be acknowledged. First, although 29 BC-PDO models were successfully established, only 21 generated complete pharmacologic datasets across all AC-T treatment conditions and could therefore be included in the comparative analyses. While this success rate is comparable to those reported in contemporary BC-PDO studies, the cohort originated from a single tertiary referral center (Sirio-Lebanese Hospital, São Paulo, BR) and therefore may not fully capture the clinical and molecular diversity of the broader BC population. Consequently, larger multicenter cohorts will be essential to validate the PCE framework and determine the generalizability of the present findings. Accordingly, the current study should be viewed primarily as an exploratory, hypothesis-generating investigation rather than a definitive validation of patient-level treatment stratification. Second, our PDO cohort was enriched for HR+/HER2− tumors (52%), whereas TNBC (13%) and HER2-positive tumors (6.5%) were underrepresented. Because taxanes show substantial clinical activity in aggressive BC subtypes, particularly TNBC and HER2-positive disease, the low prevalence of PTX-responsive PDOs observed here should be interpreted cautiously and may partly reflect cohort composition rather than a universal feature of BC. Therefore, the low prevalence of PTX-responsive PDOs observed here should not be interpreted as evidence against the broader clinical relevance of taxanes in BC. In addition, our criteria may have been conservative in distinguishing weak cytotoxic/cytostatic from predominantly inactive responses, which is relevant for borderline PTX-sensitive cases. Notably, despite the limited representation of aggressive subtypes in our cohort, the strongest PTX responses were predominantly observed in PDOs derived from TNBC (HSL-PDO#8, PCE = 89.5; HSL-PDO#16, PCE = 84.6) and HER2-positive disease (HSL-PDO#19, PCE = 73.2). Although based on few cases, this qualitative pattern is consistent with the well-established clinical activity of taxanes in these molecular contexts and further suggests that the low overall frequency of PTX-responsive PDOs in the present study is likely influenced by cohort composition rather than representing a general feature of BC biology. Nevertheless, not all aggressive tumors exhibited high PTX sensitivity. For example, HSL-PDO#12, a TNBC model (Supplementary Table S2), displayed a PD-like response (PCE = 9.7), illustrating the marked functional heterogeneity that exists even within clinically taxane-responsive subtypes. In addition, compared with monolayer cultures, PDOs preserve 3D architecture, ECM interactions, and relative lower proliferative activity, features that have been associated with increased intrinsic drug resistance and reduced taxane sensitivity in BC 3D models [34]. These characteristics may partly explain why conventional 2D cell lines often overestimate taxane responsiveness relative to clinically relevant PDO models. Moreover, taxane responsiveness is influenced by multiple biological mechanisms beyond molecular subtype. Altered microtubule dynamics, including changes in β-tubulin isoform expression, activation of ATP-binding cassette (ABC) drug-efflux transporters, EMT plasticity, and other adaptive resistance programs have all been implicated in reduced taxane sensitivity in BC. These mechanisms were not investigated in the present study but may contribute to the heterogeneous PTX response profiles observed across our PDO cohort, consistent with recent reviews of resistance biology [29].

Third, PDOs remain a simplified representation of human tumors. Although they preserve key intrinsic tumor features and interpatient heterogeneity, they do not recapitulate immune interactions, stromal influences, vascularization, systemic pharmacokinetics, or host-dependent drug metabolism. Recent advances in next-generation organoid technologies, including immune and stromal co-culture systems, organoid-on-chip platforms, and 3D bioprinting, represent promising strategies to better recapitulate the tumor microenvironment and further improve the physiological relevance and translational potential of PDO-based drug testing [15]. PDO-derived responses should therefore be interpreted as measurements of intrinsic tumor vulnerabilities rather than complete representations of treatment activity in vivo.

This limitation is especially relevant for cyclophosphamide, which requires hepatic bioactivation. Accordingly, PDO experiments employed 4-OHC, the principal active metabolite, as a surrogate for activated drug exposure. Although this approach bypasses hepatic metabolism and enables direct assessment of tumor-intrinsic sensitivity, it cannot fully reproduce the systemic pharmacology of cyclophosphamide in patients. Importantly, the heterogeneous functional activity of 4-OHC observed across the PDO cohort suggests that limited responses cannot be attributed solely to this experimental simplification. While 8 of the 21 PDOs exhibited PR-like or CR-like responses (PCE ≥ 50), 8 were classified as PD-like (PCE < 20), highlighting substantial interpatient variability in sensitivity to the active metabolite itself. This heterogeneity is consistent with known biological mechanisms of cyclophosphamide resistance, including increased aldehyde dehydrogenase (ALDH)-mediated detoxification, enhanced glutathione-dependent drug inactivation, and other intrinsic resistance pathways that reduce sensitivity to activated cyclophosphamide metabolites [35]. An additional consideration is the intrinsic chemical instability of 4-OHC itself. Under physiological aqueous conditions, 4-OHC undergoes rapid spontaneous conversion to aldophosphamide and downstream metabolites, with a reported half-life on the order of tens of minutes at 37 °C [36]. Although this chemical lability may reduce sustained drug exposure during prolonged ex vivo incubations, it is unlikely to fully explain our findings, since multiple PDOs still exhibited robust functional responses to 4-OHC, indicating that intrinsic tumor sensitivity remained readily detectable under the experimental conditions used. Furthermore, PDO models do not reproduce systemic pharmacokinetic influences or host-related determinants of cyclophosphamide activity, which may also contribute to the observed variability.

A major translational limitation is that we were unable to systematically match PDO-derived response profiles with the corresponding patients’ clinical outcomes following neoadjuvant AC-T therapy. This precludes formal assessment of the predictive value of PDO-derived response states in the clinical setting. At the same time, it is important to recognize that conventional clinical endpoints such as pathological complete response, residual cancer burden, DFS, or OS capture the integrated outcome of the full regimen and generally do not resolve the relative contribution of individual constituent drugs. Still, one anecdotal case illustrates the type of clinical-biological relationship that PDO-based analyses may uncover. HSL-PDO1, derived from a treatment-naïve HR+/HER2− invasive BC (Supplementary Table S2), showed marked sensitivity to DOXO despite profound resistance to both 4-OHC and PTX (Figure 3D). Clinically, the corresponding patient exhibited extensive residual disease after neoadjuvant AC-T, with a final classification of RCB-III, indicating poor overall response to the regimen. Although this single observation cannot support any predictive conclusion, it illustrates the central hypothesis of the present study: that a tumor classified clinically as a poor responder to AC-T may still harbor a highly asymmetric pattern of sensitivity to individual regimen components. This type of constituent-level information cannot be directly resolved through conventional clinical outcome measures alone. Importantly, this individual case should be interpreted as a qualitative proof-of-concept illustration supporting the biological plausibility of the PCE framework rather than statistically powered clinical validation of its predictive performance. Prospective studies correlating PDO-derived PCE profiles with pathological response and long-term clinical outcomes will be necessary to establish its predictive value and clinical utility.

## 5. Conclusions

In conclusion, our results support a view of AC-T in which treatment efficacy emerges from heterogeneous and partially independent drug contributions across tumors rather than from universal pharmacologic synergy within each tumor. If different patients derive benefit from different components of standard chemotherapy regimens, our findings provide an exploratory framework suggesting that the future of chemotherapy personalization may depend not only on identifying better combinations, but also on prospectively defining the minimal effective subset of drugs required for each individual tumor. Whether this concept can ultimately support chemotherapy de-escalation or other treatment modifications will require prospective clinical validation through studies correlating PDO-derived functional response profiles with pathological response and long-term clinical outcomes. In this context, PDOs represent not only a promising platform for investigating therapeutic personalization, but also a tractable experimental system for testing hypotheses regarding drug contribution, regimen design, treatment de-escalation, and the biological structure of combination chemotherapy.

## Figures and Tables

**Figure 1:**
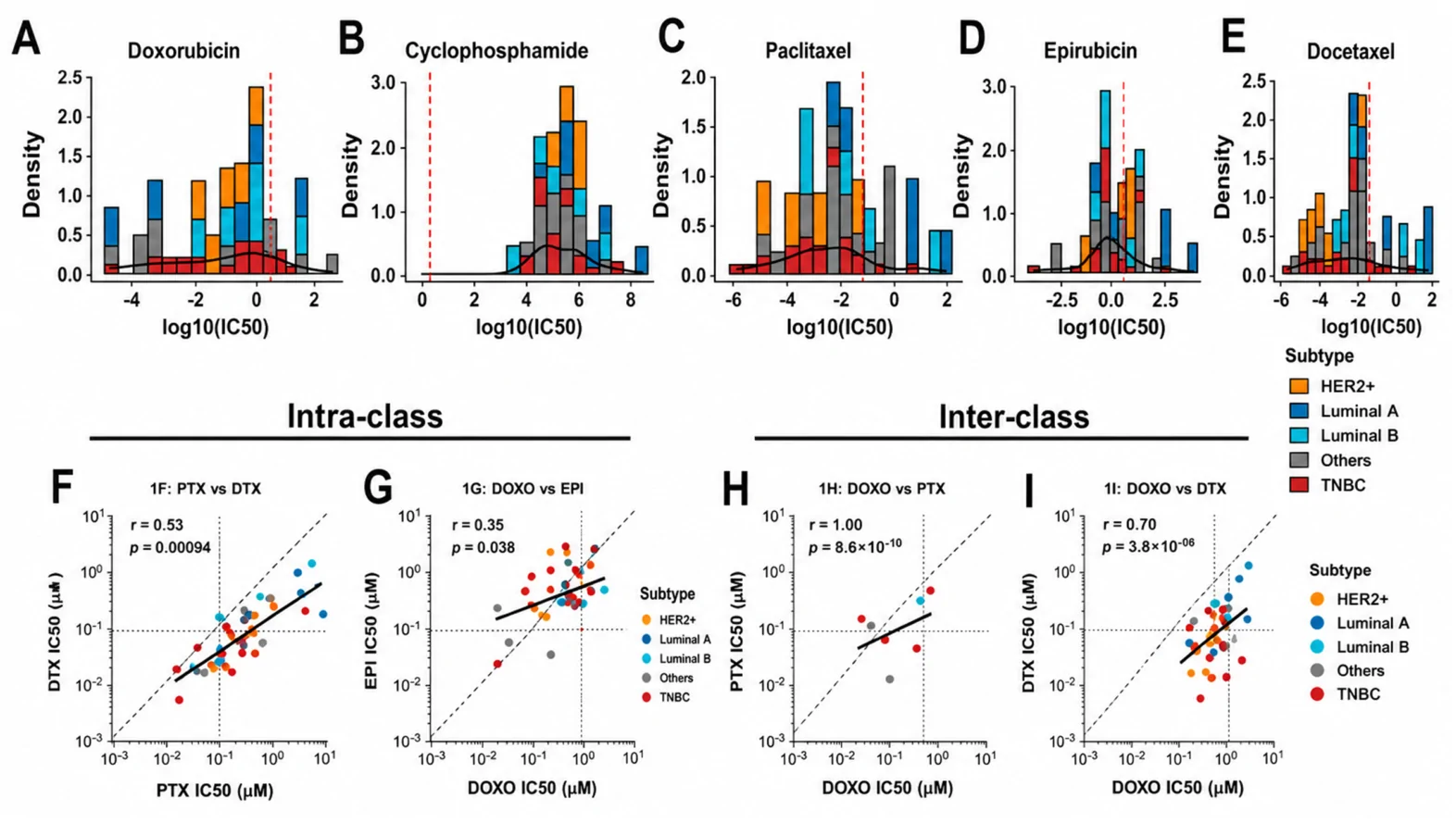
**Heterogeneous drug responses across breast cancer models.** (**A**–**E**) Distribution of IC_50_ values for doxorubicin (DOXO), cyclophosphamide (CICLO), paclitaxel (PTX), epirubicin (EPI) and docetaxel (DTX) across BC cell lines from the GDSC dataset. Histograms are overlaid with density curves and stratified by molecular subtype. Vertical dashed red lines indicate clinically achievable free drug exposure (Cmax). (**F**–**I**) Pairwise correlation of IC_50_ values for intra-class ((**F**): PTX vs. DTX; (**G**): DOXO vs. EPI) and inter-class ((**H**): DOXO vs. PTX; (**I**): DOXO vs. DTX) comparisons. Each point represents one cell line, colored by subtype. Dashed lines indicate the line of identity. Dotted lines represent Cmax thresholds, defining quadrants of sensitivity and resistance. Quadrant-based analysis revealed substantial discordance across all comparisons.

**Figure 2:**
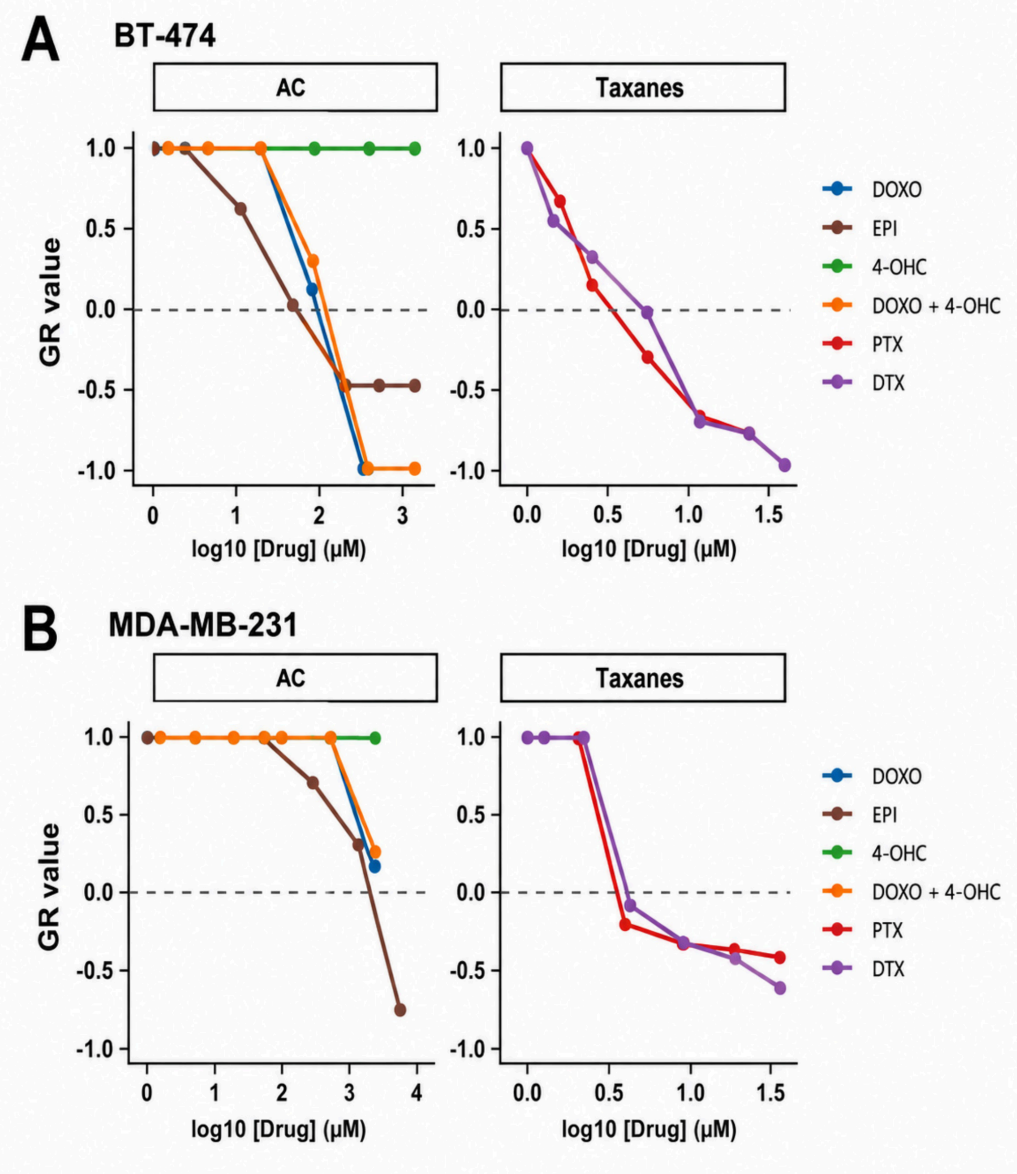
**Breast cancer cell lines reveal diferential contributions of individual AC-T components.** (**A**) GR-based dose–response curves for BT-474 cells exposed to the anthracycline/cyclophosphamide (AC) arm: doxorubicin (DOXO); 4-hydroxy cyclophosphamide (4-OHC); DOXO + 4-OHC; epirubicin (EPI) and to the taxane (T) arm: paclitaxel (PTX) and docetaxel (DTX). BT-474 displayed marked sensitivity to both A and T, whereas 4-OHC remained inactive. DOXO and DOXO + 4-OHC showed nearly superimposable curves, indicating no evident functional gain from adding 4-OHC to the anthracycline backbone. (**B**) GR-based dose–response curves for MDA-MB-231 cells. In this exploratory model, activity was concentrated predominantly in the T arm, with PTX and DTX showing the strongest responses, whereas DOXO and DOXO + 4-OHC showed limited activity and 4-OHC remained inactive.

**Figure 3:**
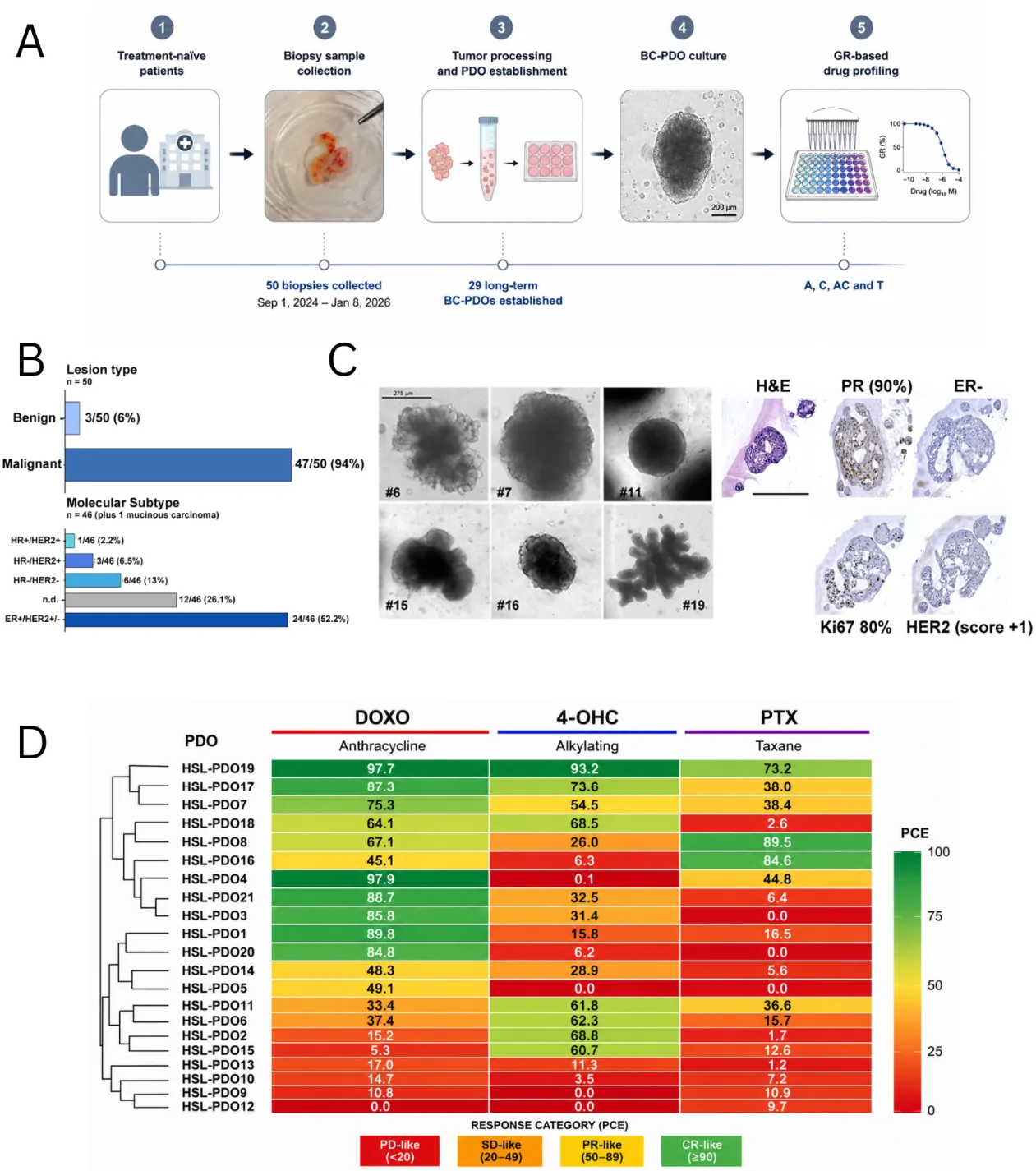
**PDOs reveal interindividual heterogeneity and non-overlapping vulnerabilities within AC-T chemotherapy.** (**A**) Workflow for BC-PDO establishment and functional profiling in HSL. Fresh biopsies were collected from treatment-naïve patients, processed for tumor dissociation and organoid establishment, expanded as BC-PDO cultures, and subjected to GR-based drug-response profiling against the components of the AC-T regimen. Scale bar = 200 µm (PDO image). (**B**) Clinical and molecular composition of the biopsy cohort. Top, distribution of benign and malignant lesions among collected biopsies. Bottom, molecular subtype distribution among malignant cases with available classification, highlighting predominance of HR+/HER2− tumors, followed by TNBC, HER2+, and one triple-positive case; a subset remained not determined (n.d.) at the time of analysis. (**C**) Morphological and pathological characterization of BC-PDOs. Left, representative bright-field images illustrating the morphological diversity of PDO cultures. Right, corresponding histopathological and immunophenotypic characterization, including H&E, PR, ER, Ki67, and HER2, demonstrating preservation of relevant tumor features in PDOs. Scale bar = 275 µm (PDO images). (**D**) Heatmap summarizing PDO responses to the individual components of the AC-T regimen based on the PCE score. PCE integrates GR50, GRmax and clinically achievable free plasma concentrations into a continuous response metric ranging from 0 to 100. Higher PCE values indicate greater pharmacological vulnerability, reflecting the combined contribution of drug potency, maximal efficacy, and clinically relevant exposure. Heatmap colors range from red (low predicted pharmacological vulnerability) to green (high predicted pharmacological vulnerability), as indicated by the accompanying color scale. Response categories were defined as PD-like (<20), SD-like (20–49), PR-like (50–89), and CR-like (≥90), corresponding to the PCE thresholds shown below the heatmap.

**Figure 4:**
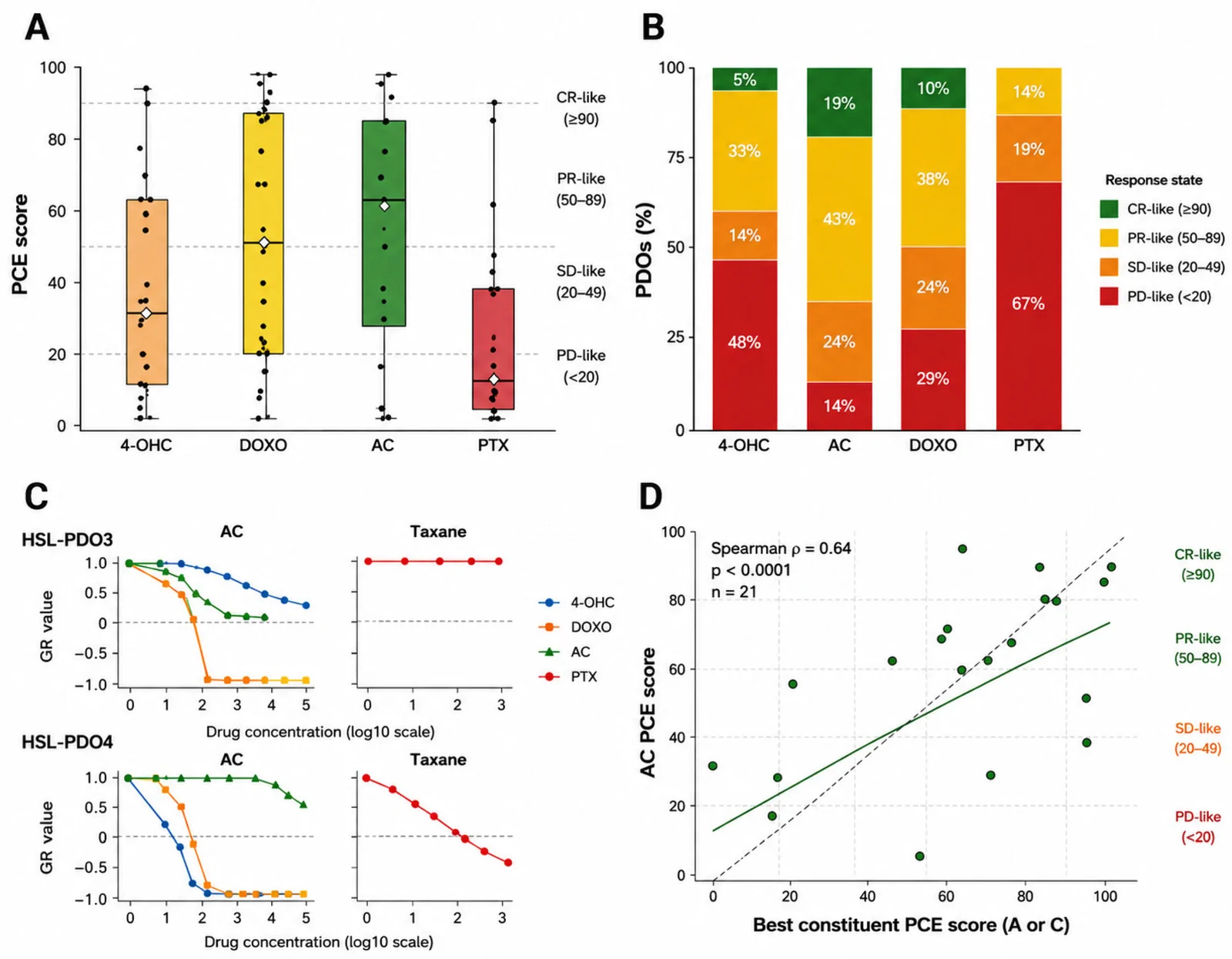
**A multidimensional PCE framework reveals heterogeneous chemotherapy response states across BC-PDOs**. (**A**) Distribution of PCE scores across treatment conditions. PCE integrates drug potency, maximal efficacy, and clinically achievable exposure into a single continuous metric ranging from 0 to 100. Each point represents an individual PDO, and the white diamond indicates the mean PCE value. Box colors identify the respective treatment conditions (4-OHC, DOXO, AC, and PTX). Horizontal lines indicate median values. (**B**) Distribution of RECIST-like response states derived from PCE scores. PDO responses were classified as Progressive Disease (PD)-like, Stable Disease (SD)-like, Partial Response (PR)-like, or Complete Response (CR)-like according to predefined PCE thresholds. AC generated the highest proportion of favorable response states, whereas PTX was predominantly associated with PD-like profiles. (**C**) Representative GR-based dose–response curves illustrating distinct patterns of chemotherapy vulnerability. HSL-PDO3 achieved a CR-like response to AC despite limited activity of 4-OHC and complete resistance to PTX, whereas HSL-PDO4 exhibited marked sensitivity to DOXO and AC while remaining unresponsive to 4-OHC. These examples illustrate how similar regimen-level responses can emerge from distinct underlying drug dependencies. Complete dose–response curves for all PDOs are provided in Supplementary Figure S1. (**D**) Relationship between AC activity and the most active constituent drug. Each point represents a PDO. The *x*-axis corresponds to the highest PCE value observed for either DOXO (**A**) or 4-OHC (**C**), and the *y*-axis represents the corresponding AC PCE value. The dashed line indicates the line of identity. The positive association between constituent and combination activity (Spearman ρ = 0.64, *p* < 0.0001) suggests that dominant single-agent vulnerabilities contribute substantially to AC efficacy, although the observed dispersion indicates that combination responses cannot be fully explained by constituent activity alone.

**Table 1:** **Classification of BC cell lines based on IC_50_ relative to clinically achievable exposure.**

Class	Drug	* Sensitive	Intermediate	Resistant
**Anthracyclines**	Doxorubicin (DOXO)	72 (36/50)	10 (5/50)	18 (9/50)
	Epirubicin (EPI)	51 (18/35)	14 (5/35)	34 (12/35)
**Taxanes**	Paclitaxel (PTX)	78 (28/36)	11 (4/36)	11 (4/36)
	Docetaxel (DTX)	64 (23/36)	11 (4/36)	25 (9/36)
	Cyclophosphamide	0 (0/50)	0 (0/50)	100 (50/50)

* Sensitivity was assessed using a simplified clinically anchored framework based on the relationship between IC_50_ and free plasma Cmax values. Models within ±30% of Cmax were classified as intermediate and excluded from binary sensitivity analyses.

**Table 2:** **Summary of GR-derived pharmacologic parameters for both models, including GR_50_, GR_max_, free Cmax, and Cmax/GR_50_ ratios.**

Cell Line	Drug	GR_50_ (µM)	GR_max_	Cmax (µM)	Cmax/GR50
BT-474	A	0.08	−1.00	1.68	21
	C	inf	1.00	1.57	inf
	AC	0.08	−1.00	1.25	16
	T	0.0016	−1.00	0.27	169
	EPI	0.064	−0.46	3.80	59
	DTX	0.0015	−1.00	0.16	107
MDA-MB-231	A	1.35	0.23	1.68	1
	C	inf	1.00	1.57	inf
	AC	1.4	0.33	1.25	1
	T	0.0025	−0.40	0.27	108
	EPI	0.74	−0.69	3.80	5
	DTX	0.0028	−0.59	0.16	57

A, doxorubicin; C, 4-hydroxy-cyclophosphamide; T, paclitaxel; EPI, epirubicin; DTX, docetaxel; inf = GR_50_ was not reached within the tested concentration range.

## Data Availability

Processed data supporting the findings of this study are available from the corresponding author upon reasonable request. Raw data derived from human samples are not publicly available due to privacy and ethical restrictions.

## Supplementary Materials

The following supporting information can be downloaded at: https://www.mdpi.com/article/10.3390/cancers18193121/s1, Figure S1: GR-based dose–response curves for the full BC-PDO cohort; Figure S2: External computational application of the Probability of Chemotherapeutic Efficacy (PCE) framework to an independent GR-based pharmacological dataset; Table S1: Pairwise statistical comparison of drug response across breast cancer cell lines; Table S2. Clinicopathological characteristics of the BC-PDO cohort included in functional analyses.

## Abbreviations

The following abbreviations are used in this manuscript:

4-OHC	4-Hydroxycyclophosphamide
AC	Doxorubicin plus 4-Hydroxycyclophosphamide
AC-T	Anthracycline/Cyclophosphamide-Taxane chemotherapy
ATCC	American Type Culture Collection
BC	Breast Cancer
BME	Basement Membrane Extract
Cmax	Maximum Plasma Concentration
CR-like	Complete Response-like
DFS	Disease-Free Survival
DOXO	Doxorubicin
DTX	Docetaxel
ECM	Extracellular matrix
EPI	Epirubicin
FBS	Fetal Bovine Serum
FDR	False Discovery Rate
GDSC	Genomics of Drug Sensitivity in Cancer
GR	Growth Rate
GR50	Concentration associated with 50% growth-rate inhibition
GRmax	Maximum growth-rate effect
HER2	Human Epidermal Growth Factor Receptor 2
HR	Hormone Receptor
IC_50_	Half-Maximal Inhibitory Concentration
IDA	Independent Drug Action
MTT	3-(4,5-Dimethylthiazol-2-yl)-2,5-Diphenyltetrazolium Bromide
OS	Overall Survival
PD-like	Progressive Disease-like
PDO	Patient-Derived Organoid
PCE	Probability of Chemotherapeutic Efficacy
PR-like	Partial Response-like
PTX	Paclitaxel
RCB	Residual Cancer Burden
RECIST	Response Evaluation Criteria in Solid Tumors
SD-like	Stable Disease-like
STR	Short Tandem Repeat
TNBC	Triple-Negative Breast Cancer
